# Supplementary material for: A mathematical model to assess the effectiveness of test-trace-isolate-and-quarantine under limited capacities
Source: PLoS One. 2024 Mar 12;19(3):e0299880. doi: 10.1371/journal.pone.0299880 (PMC10931449; doi:10.1371/journal.pone.0299880)
Supplement: S1 File — Appendix A Derivation of contact tracing terms with early and late infectious individuals, Appendix B Modeling social and hygiene measures and changes in the tracing coverage, Appendix C Parameterization, Appendix D Stability analysis. (ZIP) [file pone.0299880.s001.zip › S1_file.pdf]

Supplementary material to: A mathematical model to assess the effectiveness of test-trace-isolate-and-quarantine under limited capacities

Julian Heidecke[1,3,4*], Jan Fuhrmann[2], Maria Vittoria Barbarossa[1]

1 Frankfurt Institute for Advanced Studies, 60438 Frankfurt, Germany
2 Institute of Applied Mathematics, Heidelberg University, 69120 Heidelberg, Germany
3 Interdisciplinary Center for Scientific Computing, Heidelberg University, 69120 Heidelberg, Germany
4 Heidelberg Institute of Global Health, Heidelberg University, 69120 Heidelberg, Germany

*julian.heidecke@iwr.uni-heidelberg.de

Appendix A Derivation of contact tracing terms with early and late infectious individuals

In this section we outline the derivation of the terms $\mathrm{Tr}_E, \mathrm{Tr}_{U_1}, \mathrm{Tr}_{U_2}$ describing contact tracing in the full model (1) in the main text. We follow the same approach as outlined in the main text for the reduced model. However, we now have to distinguish between index cases detected while being in the early infectious phase U_1 and those being detected while being in the late infectious phase U_2. On average, these different types of index cases report different numbers of infected contacts, and their contacts spent a different duration in the infected chain at the time of being quarantined. Throughout the derivation we apply similar approximations as for the reduced model in the main text where we discuss the rationale behind them in more detail.

First, consider an average index case who is detected by testing at time $t-\kappa$, thus initiates contact tracing at time t, and suppose the index case is in U_2 at the time of detection (here called U_2*-index case*). Following the same reasoning as in the main text, we assume that the PHA choose a tracing window T and ask for close contacts from the tracing interval $J_T^{U_2}(t) = [t-\kappa-T, t-\kappa]$. The duration $\tau^{U_2}(t)$ for which the average U_2-index case initiating contact tracing at time t has been infectious by the time of detection $t-\kappa$ is the sum of the time spent in U_1 and the time spent in U_2,

$$\tau^{U_2}(t) = \tau_{U_1}^{U_2}(t) + \tau_{U_2}^{U_2}(t).$$

Depending on the tracing window T, the tracing interval $J_T^{U_2}(t)$ is composed of three subintervals:

- the (potentially trivial) part of $J_T^{U_2}(t)$ during which the index case was not yet infectious

$$J_T^{U_2,\mathrm{ninf}}(t) = \begin{cases} \emptyset, & \text{if } T \le \tau^{U_2}(t) \\ [t-\kappa-T, t-\kappa-\tau^{U_2}(t)], & \text{if } T > \tau^{U_2}(t) \end{cases}$$

- the (potentially trivial) part of $J_T^{U_2}(t)$ where the index case was in the early infectious phase

$$J_T^{U_2,\mathrm{early}}(t) = \begin{cases} \emptyset, & \text{if } T \le \tau_{U_2}^{U_2}(t) \\ [t-\kappa-T, t-\kappa-\tau_{U_2}^{U_2}(t)], & \text{if } \tau_{U_2}^{U_2}(t) < T \le \tau^{U_2}(t) \\ [t-\kappa-\tau^{U_2}(t), t-\kappa-\tau_{U_2}^{U_2}(t)], & \text{if } T > \tau^{U_2}(t) \end{cases} \tag{1}$$

- the time in $J_T^{U_2}(t)$ during which the index case was in the late infectious phase

$$J_T^{U_2,\mathrm{late}}(t) = \begin{cases} [t-\kappa-T, t-\kappa], & \text{if } T \le \tau_{U_2}^{U_2}(t) \\ [t-\kappa-\tau_{U_2}^{U_2}(t), t-\kappa], & \text{if } T > \tau_{U_2}^{U_2}(t). \end{cases} \tag{2}$$

In reality, the close contact definition probably leads to different reported close contact rates $\widetilde{c}_0, \widetilde{c}_1, \widetilde{c}_2$ corresponding to $J_T^{U_2,\mathrm{ninf}}(t), J_T^{U_2,\mathrm{early}}(t), J_T^{U_2,\mathrm{late}}(t)$ and different infection probabilities $\widetilde{p}_1, \widetilde{p}_2$ corresponding to $J_T^{U_2,\mathrm{early}}(t), J_T^{U_2,\mathrm{late}}(t)$. These result in transmission rates observed by contact tracing $\widetilde{\beta}_1 := \widetilde{p}_1 \widetilde{c}_1$ in $J_T^{U_2,\mathrm{early}}(t)$ and $\widetilde{\beta}_2 := \widetilde{p}_2 \widetilde{c}_2$ in $J_T^{U_2,\mathrm{late}}(t)$. Following our argumentation for the reduced model in the main text, we make the simplifying assumption that $\widetilde{c} := \widetilde{c}_0 = \widetilde{c}_1 = \widetilde{c}_2$. Additionally, we set $\widetilde{p}_1 = \theta \widetilde{p}_2$, where θ is the scaling factor for the early infectious transmission rate. Note that this results in

$$\frac{\widetilde{\beta}_1}{\beta_{U_1}} = \frac{\widetilde{\beta}_2}{\beta_{U_2}}. \tag{3}$$

Following the approach in the main text, we write the rate at which contacts of U_2-index cases become traceable at time t as

$$c_{\mathrm{pot}}^{U_2}(t) = \int_{J_T^{U_2}(t)} \widetilde{c} N_F(s)\, \eta_{U_2}\big|_{t-\kappa} U_2(t-\kappa) ds \approx \left|J_T^{U_2}(t)\right| \widetilde{c} N_F(t-\kappa)\, \eta_{U_2}\big|_{t-\kappa} U_2(t-\kappa). \tag{4}$$

where $N_F = S + E + U_1 + U_2 + \rho_Q(Q_E + Q_{U_1} + Q_{U_2}) + \rho_I(I_1 + I_2) + R$. The rate at which infected contacts of U_2-index cases become traceable at time t is the sum of those infected while their U_2-index case was in U_1

$$c_{\mathrm{pot},U_1}^{\mathrm{inf},U_2}(t) = \int_{J_T^{U_2,\mathrm{early}}(t)} \widetilde{\beta}_1\, S(s)\, \eta_{U_2}\big|_{t-\kappa} U_2(t-\kappa) ds \approx \left|J_T^{U_2,\mathrm{early}}(t)\right| \widetilde{\beta}_1\, S(t-\kappa) \eta_{U_2}\big|_{t-\kappa} U_2(t-\kappa),$$

and those infected while their U_2-index case was in U_2

$$c_{\mathrm{pot},U_2}^{\mathrm{inf},U_2}(t) = \int_{J_T^{U_2,\mathrm{late}}(t)} \widetilde{\beta}_2\, S(s)\, \eta_{U_2}\big|_{t-\kappa} U_2(t-\kappa) ds \approx \left|J_T^{U_2,\mathrm{late}}(t)\right| \widetilde{\beta}_2\, S(t-\kappa) \eta_{U_2}\big|_{t-\kappa} U_2(t-\kappa).$$

In order to later approximate the distribution of infected contacts over the infected compartments, we approximate the time that contacts that were infected while their U_2-index case was in U_1 have spent in the infected chain by the time t of being traced as

$$\widetilde{r}_{U_1}^{U_2}(t) = \kappa + \frac{1}{2}\left|J_T^{U_2,\mathrm{early}}(t)\right| + \left|J_T^{U_2,\mathrm{late}}(t)\right|. \tag{5}$$

For those contacts that were infected while their U_2-index case was in U_2 we approximate this time as

$$\widetilde{r}_{U_2}^{U_2}(t) = \kappa + \frac{1}{2}\left|J_T^{U_2,\mathrm{late}}(t)\right|. \tag{6}$$

Let us now consider an average index case who is detected at time $t-\kappa$, thus initiates contact tracing at time t, and who is in U_1 at the time of detection (U_1*-index case*). For simplicity, we assume that the same tracing window T as for U_2-index cases is chosen, thus, the index case is asked to disclose close contacts from $J_T^{U_1}(t) = [t-\kappa-T, t-\kappa]$. Let $\tau^{U_1}(t)$ denote the time the average U_1-index initiating contact tracing at time t has been infectious by the time $t-\kappa$ of being detected. Depending on the relationship between T and $\tau^{U_1}(t)$, the tracing interval $J_T^{U_1}(t)$ is composed of two subintervals:

- the potentially trivial part of $J_T^{U_1}(t)$ during which the index case was not yet infectious

$$J_T^{U_1,\mathrm{ninf}}(t) = \begin{cases} \emptyset, & \text{if } T \leq \tau^{U_1}(t) \\ [t-\kappa-T, t-\kappa-\tau^{U_1}(t)], & \text{if } T > \tau^{U_1}(t) \end{cases}$$

- the time in $J_T^{U_1}(t)$ during which the index case was in the early infectious phase

$$J_T^{U_1,\mathrm{early}}(t) = \begin{cases} [t-\kappa-T, t-\kappa], & \text{if } T \leq \tau^{U_1}(t) \\ [t-\kappa-\tau^{U_1}(t), t-\kappa], & \text{if } T > \tau^{U_1}(t). \end{cases} \tag{7}$$

We approximate the rate at which contacts of U_1-index cases become traceable at time t as

$$c_{\text{pot}}^{U_1}(t) = \int_{J_T^{U_1}(t)} \widetilde{c} N_F(s)\, \eta_{U_1}\big|_{t-\kappa} U_1(t-\kappa) ds \approx \left|J_T^{U_1}(t)\right| \widetilde{c} N_F(t-\kappa)\, \eta_{U_1}\big|_{t-\kappa} U_1(t-\kappa), \quad (8)$$

The rate at which infected contacts of U_1-index cases become traceable at time t is set to

$$\begin{aligned} c_{\text{pot}}^{\text{inf},U_1}(t) &= \int_{J_T^{U_1,\text{early}}(t)} \widetilde{\beta}_1\, S(s)\, \eta_{U_1}(t-\kappa) U_1(t-\kappa) ds \\ &\approx \left|J_T^{U_1,\text{early}}(t)\right| \widetilde{\beta}_1\, S(t-\kappa) \eta_{U_1}\big|_{t-\kappa} U_1(t-\kappa). \end{aligned}$$

The time such contacts have stayed in the infected chain by the time of contact tracing t is estimated as

$$\widetilde{r}^{U_1}(t) = \kappa + \frac{1}{2}\left|J_T^{U_1,\text{early}}(t)\right|. \quad (9)$$

The sum of Eq (4) and Eq (8) gives the total rate at which contacts become traceable at time t,

$$c_{\text{pot}}(t) = c_{\text{pot}}^{U_2}(t) + c_{\text{pot}}^{U_1}(t), \quad (10)$$

which determines the tracing efficiency (see Eq (6) in the main text)

$$\varepsilon(t) = \frac{\Omega}{\left\|(c_{\text{pot}}(t), \Omega)\right\|_p}. \quad (11)$$

The actual rates at which infected contacts are quarantined at time t are then given by

$$c_{\text{act}}^{\text{inf},U_1}(t) = c_{\text{pot}}^{\text{inf},U_1}(t)\varepsilon(t), \quad (12)$$

$$c_{\text{act},U_1}^{\text{inf},U_2}(t) = c_{\text{pot},U_1}^{\text{inf},U_2}(t)\varepsilon(t), \quad (13)$$

$$c_{\text{act},U_2}^{\text{inf},U_2}(t) = c_{\text{pot},U_2}^{\text{inf},U_2}(t)\varepsilon(t). \quad (14)$$

In order to approximate from which compartment the infected contacts described by these rates originate, we seed every infected contact in the exposed compartment and follow the first-order kinetics of system (1) in the main text for the durations (9),(5),(6) . To this end, we solve the auxiliary initial-value problem

$$\begin{aligned} \frac{d\widetilde{E}}{ds} &= -\alpha \widetilde{E} \\ \frac{d\widetilde{U}_1}{ds} &= -(\gamma_1 + \eta_{U_1}\big|_{t-\kappa})\widetilde{U}_1 + \alpha \widetilde{E} \\ \frac{d\widetilde{U}_2}{ds} &= -(\gamma_2 + \eta_{U_2}\big|_{t-\kappa})\widetilde{U}_2 + \gamma_1 \widetilde{U}_1 \\ \widetilde{E}(0) &= 1 \\ \widetilde{U}_1(0) &= 0 \\ \widetilde{U}_2(0) &= 0 \end{aligned} \quad (15)$$

and evaluate the solution at time $s = \widetilde{r}^{U_1}(t)$ when considering contacts of U_1-index cases; at time $s = \widetilde{r}_{U_1}^{U_2}(t)$ for contacts of U_2-index cases that were infected when the respective index case was in U_1; and at time $s = \widetilde{r}_{U_2}^{U_2}(t)$ when considering contacts of U_2-index cases that were infected when the respective index case was in U_2. This results in fractions $\mu_E^{U_1}(t)$ of (12), $\mu_{U_1,E}^{U_2}(t)$ of (13) and $\mu_{U_2,E}^{U_2}(t)$ of (14) originating from the E compartment. Similarly, we get fractions $\mu_{U_1}^{U_1}(t)$ of (12),

$\mu^{U_2}_{U_1,U_1}(t)$ of (13) and $\mu^{U_2}_{U_2,U_1}(t)$ of (14) originating from the U_1 compartment and lastly fractions $\mu^{U_1}_{U_2}(t)$ of (12), $\mu^{U_2}_{U_1,U_2}(t)$ of (13) and $\mu^{U_2}_{U_2,U_2}(t)$ of (14) originating from the U_2 compartment. Notice that in order to simplify solving solutions to the auxiliary model (15) we have neglected changes in the testing rates η_{U_1}, η_{U_2} over the infectious phases of the tracing intervals.

We end up with the following terms describing contact tracing at time t

$$\mathrm{Tr}_E(t) = \mu^{U_1}_E(t)c^{\mathrm{inf},U_1}_{\mathrm{act}}(t) + \mu^{U_2}_{U_1,E}(t)c^{\mathrm{inf},U_2}_{\mathrm{act},U_1}(t) + \mu^{U_2}_{U_2,E}(t)c^{\mathrm{inf},U_2}_{\mathrm{act},U_2}(t), \tag{16}$$

$$\mathrm{Tr}_{U_1}(t) = \mu^{U_1}_{U_1}(t)c^{\mathrm{inf},U_1}_{\mathrm{act}}(t) + \mu^{U_2}_{U_1,U_1}(t)c^{\mathrm{inf},U_2}_{\mathrm{act},U_1}(t) + \mu^{U_2}_{U_2,U_1}(t)c^{\mathrm{inf},U_2}_{\mathrm{act},U_2}(t), \tag{17}$$

$$\mathrm{Tr}_{U_2}(t) = \mu^{U_1}_{U_2}(t)c^{\mathrm{inf},U_1}_{\mathrm{act}}(t) + \mu^{U_2}_{U_1,U_2}(t)c^{\mathrm{inf},U_2}_{\mathrm{act},U_1}(t) + \mu^{U_2}_{U_2,U_2}(t)c^{\mathrm{inf},U_2}_{\mathrm{act},U_2}(t). \tag{18}$$

In the setting of this model we get three different tracing coverages for the three different types of secondary infections. The tracing coverage for individuals who got infected by U_1-index cases is given by

$$\omega^{U_1}(t) := \frac{\left|J^{U_1,\mathrm{early}}_T(t)\right|}{\tau^{U_1}(t)} \frac{\widetilde{\beta}_1}{\beta_{U_1}}. \tag{19}$$

The tracing coverage for individuals who got infected by U_2-index cases when these respective index cases were in U_1, is given by

$$\omega^{U_2}_{U_1}(t) := \frac{\left|J^{U_2,\mathrm{early}}_T(t)\right|}{\tau^{U_2}_{U_1}(t)} \frac{\widetilde{\beta}_1}{\beta_{U_1}}. \tag{20}$$

Lastly, the tracing coverage for individuals who got infected by U_2-index cases when these respective index cases were in U_2, is given by

$$\omega^{U_2}_{U_2}(t) := \frac{\left|J^{U_2,\mathrm{late}}_T(t)\right|}{\tau^{U_2}_{U_2}(t)} \frac{\widetilde{\beta}_2}{\beta_{U_2}}. \tag{21}$$

We could continue with this scheme and divide the infectious period into more subperiods. In this way, we could approximate a realistic infectivity profile along the infectious period. Alternatively, a more elegant approach would be to continuously structure the infected compartment by age of infection, thereby overcoming the limitation to consider average index cases and estimation of their expected age of infection [1–7]. However, the approach presented here is sufficient to illustrate the adverse effect of transmission prior to the occurrence of potential symptoms on the effectiveness of TTIQ.

Appendix B Modeling social and hygiene measures and changes in the tracing coverage

In this section we discuss how we account for changes in social and hygiene measures (i.e., non constant transmission rates), as well as measures affecting the tracing coverage (e.g., changes in the close contact definition or an increasing awareness to keep track of personal contacts), in the contact tracing terms. As for our derivation of the contact tracing terms in the main text, for simplicity, we explain our approach and assumptions by means of a reduced model without decomposed infectious compartments into early and late infectious phase. We proceeded similarly for the full model (1) in the main text and briefly comment on this case at the end of this section.

We assume that changes to the contact tracing scheme affecting the tracing coverage deviate the contact tracing parameters discontinuously with respect to different "generations" of index cases. Specifically, changes taking place from time t on affect only the contact tracing executed on index

cases that were detected at time $t-\kappa$ or later, even though these might have overlapping tracing intervals with earlier detected index cases. We indicate this time-dependency of the contact tracing parameters by writing $\widetilde{p}_t, \widetilde{c}_t, \widetilde{\beta}_t$ for the infection probability, the contact rate and the transmission rate observed by contact tracing, respectively. We refer to this as the contact tracing scheme applied to contacts being traced at time t. Accordingly, the rate at which contacts become traceable at time t reads (coincides with Eq (4) in the main text)

$$c_{\text{pot}}(t) = \int_{J_T(t)} \widetilde{c}_t N_F(s)\, \eta_U\big|_{t-\kappa} U(t-\kappa) ds \approx |J_T(t)|\, \widetilde{c}_t N_F(t-\kappa)\, \eta_U\big|_{t-\kappa} U(t-\kappa), \tag{22}$$

and the rate at which infected contacts are quarantined at time t is given by (coincides with Eq (7) in the main text)

$$c_{\text{act}}^{\text{inf}}(t) = \int_{J_T^{\text{inf}}(t)} \widetilde{\beta}_t\, S(s) \eta_U\big|_{t-\kappa} U(t-\kappa)\varepsilon(t) ds \approx \left|J_T^{\text{inf}}(t)\right| \widetilde{\beta}_t\, S(t-\kappa)\eta_U\big|_{t-\kappa} U(t-\kappa)\varepsilon(t). \tag{23}$$

As discussed in the main text, changes in the transmission rates are modeled by a time dependent factor $\phi(t) \in [0,1]$, so that

$$\beta_U(t) = \phi(t)\overline{\beta_U},$$

where $\overline{\beta_U}$ is the baseline transmission rate of the disease corresponding to a phase without any intervention. When there is a reduction in transmission rate in place at some time point t^* so that $\beta_U(t^*) = \phi(t^*)\overline{\beta_U}$, this potentially also affects $\widetilde{\beta}_t, \widetilde{p}_t, \widetilde{c}_t$ along their relevant time intervals $J_T(t)$ and $J_T^{\text{inf}}(t)$, for all t for which $t^* \in J_T(t)$. To account for this, we let $\overline{\widetilde{p}_t}, \overline{\widetilde{c}_t}$ denote the infection probability and contact rate observed by contact tracing corresponding to a phase without any intervention in combination with the specific contact tracing scheme applied to contacts traced at time t. We express deviations from these baseline values due to social and hygiene measures by $\widetilde{\phi}_{1,t}(s)$ and $\widetilde{\phi}_{2,t}(s)$, such that

$$\begin{aligned} \widetilde{c}_t(s) &= \widetilde{\phi}_{1,t}(s)\overline{\widetilde{c}_t}, \quad s \in J_T(t), \\ \widetilde{p}_t(s) &= \widetilde{\phi}_{2,t}(s)\overline{\widetilde{p}_t}, \quad s \in J_T^{\text{inf}}(t), \\ \widetilde{\beta}_t(s) &= \widetilde{p}_t(s)\widetilde{c}_t(s), \quad s \in J_T^{\text{inf}}(t), \end{aligned}$$

whereby given a time $s \in J_T^{\text{inf}}(t)$ we must have $\widetilde{\beta}_t(s) \leq \beta(s)$. The quantities $\widetilde{\phi}_{1,t}(s)$ and $\widetilde{\phi}_{2,t}(s)$ are influenced by the contact tracing scheme applied at time t indicated by the index t and by the social and hygiene measures that where in place during the time index cases are asked to disclose contacts from, indicated by the argument s. Their values could be derived from detailed index case and contact tracing data. However, in this work we confine ourselves to the simplified (non-generic) setting where

$$\begin{aligned} \widetilde{\phi}_{1,t}(s) &= \phi(s), \quad \forall\, t \text{ and } \forall\, s \in J_T(t), \\ \widetilde{\phi}_{2,t}(s) &= 1, \quad \forall\, t \text{ and } \forall\, s \in J_T^{\text{inf}}(t). \end{aligned} \tag{24}$$

This resembles that a reduction in general transmission rates by the factor ϕ during the tracing interval $J_T(t)$ reduces contact rates observed by contact tracing at time t by the same factor, independent of the contact tracing scheme applied at time t. However, the corresponding infection probabilities are assumed to be unaffected by the particular value of ϕ. Note that the introduction of time-varying social and hygiene measures makes $\widetilde{c}_t$ and $\widetilde{\beta}_t$ dependent on the integration variable in Eq (22) and Eq (23). To circumvent this, we further simplify our assumptions by setting $\widetilde{c}_t \equiv \widetilde{c}_t(t-\kappa)$ over $J_T(t)$ ($\widetilde{p}_t$ is constant on $J_T^{\text{inf}}(t)$ by Eq (24)). This only introduces a minor deviation from the intended setting (24) since we only consider single parameter change points in our simulations, meaning that $T = |J_T(t)|$ is reasonably small compared to intervals between parameter changes (where ϕ is constant). This leads to

$$\omega(t) = \frac{\left|J_T^{\text{inf}}(t)\right|}{\tau(t)} \frac{\widetilde{\beta}_t(t-\kappa)}{\beta_U(t-\kappa)}$$

being our proxy for the tracing coverage at time t.

The discussed assumptions extend to the model with early and late infectious phase (1) in the main text. Most importantly, we assume that changes to the tracing scheme equally increase or decrease the different tracing coverages (19)-(21), and that changes in the transmission rates β_{U_2}, $\beta_{U_1} = \theta\beta_{U_2}$ affect contact rates observed by contact tracing by the same factor but do not affect the corresponding infection probabilities observed by contact tracing (as in Eq (24)).

Appendix C Parameterization

Our baseline parameter setting is inspired by the spread of COVID-19 in Germany in late summer and fall of 2020. All scenarios considered in the main text in which parameter values deviate from this baseline setting are clearly indicated and explained there.

Parameters describing spreading dynamics and disease characteristics are based on literature dedicated to the original strain of SARS-CoV-2 circulating in 2020. We assume a latent phase of about $1/\alpha = 3.5$ days [8]. This latent phase is followed by an early infectious phase of about $1/\gamma_1 = 2$ days [9], which together with the latent period of about 3.5 days gives an incubation period of about 5.5 days for individuals with a symptomatic course of infection [10–12]. We assume that individuals go trough an infectious phase of in total about 9 days and accordingly set $1/\gamma_2 = 7$ [9, 12–15]. The baseline transmission rate in the late infectious compartment is chosen as $\overline{\beta_{U_2}} = 0.33/N$. In addition, we set the scaling factor for the transmission rate of early infectious infectious individuals to $\theta = 1.5$. At low prevalence, this means that approximately 40% [1, 9, 12, 16] of transmissions from undetected infectious individuals originate from the early phase of infection and gives a basic reproduction number of $\mathcal{R}_0 = ((\theta\overline{\beta_{U_2}})/\gamma_1 + \overline{\beta_{U_2}}/\gamma_2)N = 3.3$ [12, 17–19].

For the maximal number of tests that can be administered and evaluated per day we only consider polymerase chain reaction tests as rapid antigen tests were not yet widely available in 2020 and assume a baseline value of $\sigma_+ = 200\,000$. Notice that we do not consider the additional effort generated by testing uninfected traced individuals, the fact that already confirmed cases might be tested repeatedly, or the possibility that all individuals might seek more testing at higher prevalence which would lead to a slower increase in the test positive rate. Therefore, we choose a relatively low value for σ_+ when compared to the theoretical testing capacity reported for Germany in late summer and fall of 2020 [20]. The test loss constant is set to $\sigma_- = 1.353\,N$ such that at low prevalence only approximately 85 000 tests are conducted per day. This roughly aligns with the reports for Germany during summer of 2021 at which time the reported incidence was indeed low [20]. It should be noticed, however, that the test capacity in 2021 was significantly increased compared to the considered period of late summer and fall of 2020. Nevertheless, our rough approach captures the detrimental effect of an increasing incidence on the index case detection rates. The relative frequencies (σ_{U_2}, σ_Q) of testing undetected late infectious and traced individuals compared to susceptibles depend on the considered disease, the testing strategy and the willingness of infectious and suspected individuals to get tested. Here we estimate that $(\sigma_{U_2}, \sigma_Q) = (93, 300)$. Individuals in all the other compartments (importantly also those in U_1) are assumed to have no increased chance of being tested when compared to a susceptible individual. The choice $\sigma_{U_2} = 93$ leads to a conservative case detection ratio of about 40% at low prevalence by testing alone. For simplicity, we choose to work with a constant tracing window $T = 1/\gamma_1 + 1/\gamma_2$, which reflects the theoretical decision to trace contacts of an index case for the average duration of the infectious period. We assume that the tracing scheme leads to a baseline value of $\overline{\widetilde{c}} = 0.8/N$ for the close contact rate reported by an interviewed index case when no contact restrictions are in place. Together with accordingly selected infection probabilities this results in a tracing coverage of $\omega = 0.65$ (below we discuss why we do not have to give different values to the different tracing coverages (19)-(21)). The maximal rate at which contacts can be quarantined per day is assumed to be $\Omega = 40\,000$. Moreover, we assume a tracing delay of $\kappa = 2$ days and a tracing efficiency constant of $p = 2$ (a higher p appears to be unrealistic considering the 400 locally managed public health departments in Germany). The total population size is set to $N = 83\,000\,000$ corresponding to the

German population as of 2020.

Our parameter choices are summarized in Table 2 in the main text. Due to a lack of detailed contact tracing data for Germany and an unknown case detection ratio, our choices for the TTIQ parameters come with significant uncertainty. However, in the main text sensitivity of our results to these parameters and alternative parameter constellations are investigated. In addition, while we are aware that parameters change much more dynamically in reality, we keep them constant in the considered simulations, apart from single change points.

Tracing terms resulting from the baseline parameter choices

Here we specify the exact form of the different components included in the contact tracing terms $\mathrm{Tr}_E(t)$, $\mathrm{Tr}_{U_1}(t)$, $\mathrm{Tr}_{U_2}(t)$ in model (1) in the main text given our above assumptions on parameter choices and the assumptions made in Appendix B.

We approximate the periods that the different types of index cases (introduced in Appendix A) spent in U_1 and U_2 before they were identified and isolated at time $t-\kappa$ as

$$\tau^{U_1}(t) = \tau^{U_2}_{U_1}(t) = \frac{1}{\eta_{U_1}\big|_{t-\kappa} + \gamma_1}, \quad \tau^{U_2}_{U_2}(t) = \frac{1}{\eta_{U_2}\big|_{t-\kappa} + \gamma_2}, \tag{25}$$

which, considering Eq (1), Eq (2) and Eq (7), together with the tracing window $T = 1/\gamma_1 + 1/\gamma_2$, leads to

$$\begin{aligned} \left|J_T^{U_1}(t)\right| &= \left|J_T^{U_2}(t)\right| = \frac{1}{\gamma_1} + \frac{1}{\gamma_2}, \\ \left|J_T^{U_1,\mathrm{early}}(t)\right| &= \left|J_T^{U_2,\mathrm{early}}(t)\right| = \frac{1}{\eta_{U_1}\big|_{t-\kappa} + \gamma_1}, \\ \left|J_T^{U_2,\mathrm{late}}(t)\right| &= \frac{1}{\eta_{U_2}\big|_{t-\kappa} + \gamma_2}. \end{aligned} \tag{26}$$

Using Eq (10), Eq (12)-(14), Eq (9), Eq (5), and Eq (6), while respecting the assumptions in Appendix B, we get

$$c_{\mathrm{pot}}(t) = \left(\frac{1}{\gamma_1} + \frac{1}{\gamma_2}\right) \widetilde{c}_t(t-\kappa) N_F(t-\kappa) \left[\eta_{U_1}\big|_{t-\kappa} U_1(t-\kappa) + \eta_{U_2}\big|_{t-\kappa} U_2(t-\kappa)\right], \tag{27}$$

$$c^{\mathrm{inf},U_1}_{\mathrm{act}}(t) = \frac{\widetilde{\beta}_{t,1}(t-\kappa)}{\eta_{U_1}\big|_{t-\kappa} + \gamma_1} S(t-\kappa)\eta_{U_1}\big|_{t-\kappa} U_1(t-\kappa)\varepsilon(t), \tag{28}$$

$$c^{\mathrm{inf},U_2}_{\mathrm{act},U_1}(t) = \frac{\widetilde{\beta}_{t,1}(t-\kappa)}{\eta_{U_1}\big|_{t-\kappa} + \gamma_1} S(t-\kappa)\eta_{U_2}\big|_{t-\kappa} U_2(t-\kappa)\varepsilon(t), \tag{29}$$

$$c^{\mathrm{inf},U_2}_{\mathrm{act},U_2}(t) = \frac{\widetilde{\beta}_{t,2}(t-\kappa)}{\eta_{U_2}\big|_{t-\kappa} + \gamma_2} S(t-\kappa)\eta_{U_2}\big|_{t-\kappa} U_2(t-\kappa)\varepsilon(t), \tag{30}$$

$$\widetilde{r}^{U_1}(t) = \kappa + \frac{1}{2}\frac{1}{\eta_{U_1}\big|_{t-\kappa} + \gamma_1}, \tag{31}$$

$$\widetilde{r}^{U_2}_{U_1}(t) = \kappa + \frac{1}{2}\frac{1}{\eta_{U_1}\big|_{t-\kappa} + \gamma_1} + \frac{1}{\eta_{U_2}\big|_{t-\kappa} + \gamma_2}, \tag{32}$$

$$\widetilde{r}^{U_2}_{U_2}(t) = \kappa + \frac{1}{2}\frac{1}{\eta_{U_2}\big|_{t-\kappa} + \gamma_2}. \tag{33}$$

Considering Eq (19)-(21), the approximations (25) and (26), together with assumption (3) and the assumptions in Appendix B, lead to a single tracing coverage

$$\omega(t) := \omega^{U_1}(t) = \omega^{U_2}_{U_1}(t) = \frac{\widetilde{\beta}_{t,1}(t-\kappa)}{\beta_{U_1}(t-\kappa)} = \frac{\widetilde{\beta}_{t,2}(t-\kappa)}{\beta_{U_2}(t-\kappa)} = \omega^{U_2}_{U_2}(t).$$

Eq (27)-(33) provide all the components necessary to calculate the tracing terms (16)-(18).

Appendix D Stability analysis

The stability of the DFE is approached by considering the linearization about the DFE of system (1) in the main text [21]. Our approximation of contact tracing with terms containing a single constant delay allows a straightforward analysis. The linearization of a system of delay differential equations with a single constant delay takes the form

$$x'(t) = Ax(t) - Bx(t-\kappa) \tag{34}$$

with matrices A and B depending on the parameters of the considered system. We consider the linearization of model (1) in the main text with respect to the variables $x = (E, Q_E, U_1, Q_{U_1}, I_1, U_2, Q_{U_2}, I_2)$. It is unnecessary to consider R and S here, since the equation for R is encoded in the remaining state variables and the equation for S only gives a zero eigenvalue since all disease free states are equilibrium solutions. The corresponding matrices are given by

$$A = \begin{bmatrix} -\alpha & 0 & \beta_{U_1}N & \rho_Q\beta_{U_1}N & \rho_I\beta_{U_1}N & \beta_{U_2}N & \rho_Q\beta_{U_2}N & \rho_I\beta_{U_2}N \\ 0 & -\alpha & 0 & 0 & 0 & 0 & 0 & 0 \\ \alpha & 0 & -(\overline{\eta_{U_1}}+\gamma_1) & 0 & 0 & 0 & 0 & 0 \\ 0 & \alpha & 0 & -(\overline{\eta_{Q_{U_1}}}+\gamma_1) & 0 & 0 & 0 & 0 \\ 0 & 0 & \overline{\eta_{U_1}} & \overline{\eta_{Q_{U_1}}} & -\gamma_1 & 0 & 0 & 0 \\ 0 & 0 & \gamma_1 & 0 & 0 & -(\overline{\eta_{U_2}}+\gamma_2) & 0 & 0 \\ 0 & 0 & 0 & \gamma_1 & 0 & 0 & -(\overline{\eta_{Q_{U_2}}}+\gamma_2) & 0 \\ 0 & 0 & 0 & 0 & \gamma_1 & \overline{\eta_{U_2}} & \overline{\eta_{Q_{U_2}}} & -\gamma_2 \end{bmatrix},$$

$$B = \begin{bmatrix} 0 & 0 & -\chi_E^{U_1} & 0 & 0 & -\chi_E^{U_2} & 0 & 0 \\ 0 & 0 & \chi_E^{U_1} & 0 & 0 & \chi_E^{U_2} & 0 & 0 \\ 0 & 0 & -\chi_{U_1}^{U_1} & 0 & 0 & -\chi_{U_1}^{U_2} & 0 & 0 \\ 0 & 0 & \chi_{U_1}^{U_1} & 0 & 0 & \chi_{U_1}^{U_2} & 0 & 0 \\ 0 & 0 & 0 & 0 & 0 & 0 & 0 & 0 \\ 0 & 0 & -\chi_{U_2}^{U_1} & 0 & 0 & -\chi_{U_2}^{U_2} & 0 & 0 \\ 0 & 0 & \chi_{U_2}^{U_1} & 0 & 0 & \chi_{U_2}^{U_2} & 0 & 0 \\ 0 & 0 & 0 & 0 & 0 & 0 & 0 & 0 \end{bmatrix},$$

where the entries

$$\begin{aligned} \chi_E^{U_1} &= \frac{\widetilde{\beta}_1}{\overline{\eta_{U_1}}+\gamma_1} N\,\overline{\eta_{U_1}}\,\mu_E^{U_1}\big|_{\mathrm{DFE}}, \\ \chi_E^{U_2} &= \frac{\widetilde{\beta}_1}{\overline{\eta_{U_1}}+\gamma_1} N\,\overline{\eta_{U_2}}\,\mu_{U_1,E}^{U_2}\big|_{\mathrm{DFE}} + \frac{\widetilde{\beta}_2}{\overline{\eta_{U_2}}+\gamma_2} N\,\overline{\eta_{U_2}}\,\mu_{U_2,E}^{U_2}\big|_{\mathrm{DFE}}, \\ \chi_{U_1}^{U_1} &= \frac{\widetilde{\beta}_1}{\overline{\eta_{U_1}}+\gamma_1} N\,\overline{\eta_{U_1}}\,\mu_{U_1}^{U_1}\big|_{\mathrm{DFE}}, \\ \chi_{U_1}^{U_2} &= \frac{\widetilde{\beta}_1}{\overline{\eta_{U_1}}+\gamma_1} N\,\overline{\eta_{U_2}}\,\mu_{U_1,U_1}^{U_2}\big|_{\mathrm{DFE}} + \frac{\widetilde{\beta}_2}{\overline{\eta_{U_2}}+\gamma_2} N\,\overline{\eta_{U_2}}\,\mu_{U_2,U_1}^{U_2}\big|_{\mathrm{DFE}}, \\ \chi_{U_2}^{U_1} &= \frac{\widetilde{\beta}_1}{\overline{\eta_{U_1}}+\gamma_1} N\,\overline{\eta_{U_1}}\,\mu_{U_2}^{U_1}\big|_{\mathrm{DFE}}, \\ \chi_{U_2}^{U_2} &= \frac{\widetilde{\beta}_1}{\overline{\eta_{U_1}}+\gamma_1} N\,\overline{\eta_{U_2}}\,\mu_{U_1,U_2}^{U_2}\big|_{\mathrm{DFE}} + \frac{\widetilde{\beta}_2}{\overline{\eta_{U_2}}+\gamma_2} N\,\overline{\eta_{U_2}}\,\mu_{U_2,U_2}^{U_2}\big|_{\mathrm{DFE}}, \end{aligned}$$

are the result of linearizing the tracing terms (16)-(18) with respect to U_1 and U_2 (note (27)-(33)) and evaluating the result at the DFE. By $\overline{\eta_X}$ we denote the detection rate in compartment X evaluated at the DFE.

The qualitative behavior of solutions of system (34) is determined by the roots of the characteristic equation of (34) given by

$$\det(-\lambda I + A + e^{-\lambda\kappa}B) = 0. \quad (35)$$

In order to numerically approximate solutions of (35) a discretization of the PDE-representation of the DDE can be used. This discretization results in a matrix whose eigenvalues are approximations for solutions of (35). To this end, we followed the discretization scheme based on a Chebyshev nodes described in [22]. The necessary code presented in [22] and [23] was – like all numerical experiments in this work – implemented in Python [24–28]. We calculated the critical level ϕ^* of effective contacts by scanning for the threshold level in effective contacts which determines a stability switch. In other words, for $\phi < \phi^*$ Eq (35) has only solutions with negative real part and the DFE is stable. For $\phi > \phi^*$ there is at least one solution of (35) with positive real part and the DFE is unstable.

References

1. L. Ferretti, C. Wymant, M. Kendall, L. Zhao, A. Nurtay, L. Abeler-Dörner, et al., Quantifying SARS-CoV-2 transmission suggests epidemic control with digital contact tracing, *Science*, **368** (2020), eabb6936. https://doi.org/10.1126/science.abb6936

2. C. Fraser, S. Riley, R. M. Anderson, N. M. Ferguson, Factors that make an infectious disease outbreak controllable, *Proceedings of the National Academy of Sciences*, **101** (2004), 6146–6151. https://doi.org/10.1073/pnas.0307506101

3. X. Huo, Modeling of contact tracing in epidemic populations structured by disease age, *Discrete & Continuous Dynamical Systems - B*, **20** (2015), 1685–1713. http://dx.doi.org/10.3934/dcdsb.2015.20.1685

4. J. Müller, B. Koopmann, The effect of delay on contact tracing, *Mathematical Biosciences*, **282** (2016), 204–214. https://doi.org/10.1016/j.mbs.2016.10.010

5. J. Müller, M. Kretzschmar, K. Dietz, Contact tracing in stochastic and deterministic epidemic models, *Mathematical Biosciences*, **164** (2000), 39–64. https://doi.org/10.1016/s0025-5564(99)00061-9

6. T. R. Pollmann, S. Schönert, J. Müller, J. Pollmann, E. Resconi, C. Wiesinger, et al., The impact of digital contact tracing on the SARS-CoV-2 pandemic—a comprehensive modelling study, *EPJ Data Science*, **10** (2021), 37. https://doi.org/10.1140/epjds/s13688-021-00290-x

7. F. Scarabel, L. Pellis, N. H. Ogden, J. Wu, A renewal equation model to assess roles and limitations of contact tracing for disease outbreak control, *Royal Society Open Science*, **8** (2021), 202091. https://doi.org/10.1098/rsos.202091

8. R. Li, S. Pei, B. Chen, Y. Song, T. Zhang, W. Yang, et al., Substantial undocumented infection facilitates the rapid dissemination of novel coronavirus (SARS-CoV-2), *Science*, **368** (2020), 489–493. https://doi.org/10.1126/science.abb3221

9. X. He, E. H. Y. Lau, P. Wu, X. Deng, J. Wang, X. Hao, et al., Temporal dynamics in viral shedding and transmissibility of COVID-19, *Nature Medicine*, **26** (2020), 672–675. https://doi.org/10.1038/s41591-020-0869-5

10. S. A. Lauer, K. H. Grantz, Q. Bi, F. K. Jones, Q. Zheng, H. R. Meredith, et al., The Incubation Period of Coronavirus Disease 2019 (COVID-19) From Publicly Reported Confirmed Cases: Estimation and Application, *Annals of Internal Medicine*, **172** (2020), 577–582. https://doi.org/10.7326/m20-0504

11. Q. Li, X. Guan, P. Wu, X. Wang, L. Zhou, Y. Tong, et al., Early Transmission Dynamics in Wuhan, China, of Novel Coronavirus–Infected Pneumonia, *New England Journal of Medicine*, **382** (2020), 1199–1207. https://doi.org/10.1056/nejmoa2001316

12. *Robert Koch Institute*, Epidemiologischer Steckbrief zu SARS-CoV-2 und COVID-19, Last accessed on 11/22/2022. Available from: https://www.rki.de/DE/Content/InfAZ/N/Neuartiges_Coronavirus/Steckbrief.html.

13. J. Bullard, K. Dust, D. Funk, J. E. Strong, D. Alexander, L. Garnett, et al., Predicting Infectious Severe Acute Respiratory Syndrome Coronavirus 2 From Diagnostic Samples, *Clinical Infectious Diseases*, **71** (2020), 2663–2666. https://doi.org/10.1093/cid/ciaa638

14. A. Singanayagam, M. Patel, A. Charlett, J. L. Bernal, V. Saliba, J. Ellis, et al., Duration of infectiousness and correlation with RT-PCR cycle threshold values in cases of COVID-19, England, January to May 2020, *Eurosurveillance*, **25** (2020), 2001483. https://doi.org/10.2807/1560-7917.es.2020.25.32.2001483

15. R. Wölfel, V. M. Corman, W. Guggemos, M. Seilmaier, S. Zange, M. A. Müller, et al., Virological assessment of hospitalized patients with COVID-2019, *Nature*, **581** (2020), 465–469. https://doi.org/10.1038/s41586-020-2196-x

16. T. Ganyani, C. Kremer, D. Chen, A. Torneri, C. Faes, J. Wallinga, et al., Estimating the generation interval for coronavirus disease (COVID-19) based on symptom onset data, March 2020, *Eurosurveillance*, **25** (2020), 2000257. https://doi.org/10.2807/1560-7917.es.2020.25.17.2000257

17. Y. Alimohamadi, M. Taghdir, M. Sepandi, Estimate of the Basic Reproduction Number for COVID-19: A Systematic Review and Meta-analysis, *Journal of Preventive Medicine and Public Health*, **53** (2020), 151–157. https://doi.org/10.3961/jpmph.20.076

18. M. A. Billah, M. M. Miah, M. N. Khan, Reproductive number of coronavirus: A systematic review and meta-analysis based on global level evidence, *PLOS ONE*, **15** (2020), e0242128. https://doi.org/10.1371/journal.pone.0242128

19. S. Zhao, Q. Lin, J. Ran, S. S. Musa, G. Yang, W. Wang, et al., Preliminary estimation of the basic reproduction number of novel coronavirus (2019-nCoV) in China, from 2019 to 2020: A data-driven analysis in the early phase of the outbreak, *International Journal of Infectious Diseases*, **92** (2020), 214–217. https://doi.org/10.1016/j.ijid.2020.01.050

20. *Robert Koch Institute*, Erfassung der SARS-CoV-2-Testzahlen in Deutschland, Last accessed on 11/22/2022. Available from: https://www.rki.de/DE/Content/InfAZ/N/Neuartiges_Coronavirus/Testzahl.html.

21. Y. Kuang, *Delay Differential Equations: With Applications in Population Dynamics*, Academic press, 1993.

22. E. Jarlebring, Some numerical methods to compute the eigenvalues of a time-delay system using Matlab, *The delay e-letter*, **2** (2008), 155.

23. L. N. Trefethen, *Spectral Methods in MATLAB*, Society for Industrial and Applied Mathematics, 2000.

24. C. R. Harris, K. J. Millman, S. J. van der Walt, R. Gommers, P. Virtanen, D. Cournapeau, et al., Array programming with NumPy, *Nature*, **585** (2020), 357–362. https://doi.org/10.1038/s41586-020-2649-2

25. P. Virtanen, R. Gommers, T. E. Oliphant, M. Haberland, T. Reddy, D. Cournapeau, et al., SciPy 1.0: fundamental algorithms for scientific computing in Python, *Nature Methods*, **17** (2020), 261–272. https://doi.org/10.1038/s41592-019-0686-2

26. J. D. Hunter, Matplotlib: A 2D Graphics Environment, *Computing in Science & Engineering*, **9** (2007), 90–95. https://doi.org/10.1109/MCSE.2007.55

27. ddeint Developer, ddeint,Version 0.2 (2019). https://pypi.org/project/ddeint/

28. R. Vallat, Pingouin: statistics in Python, *Journal of Open Source Software*, **3** (2018), 1026. https://doi.org/10.21105/joss.01026
